# Management of cesarean scar pregnancy: Importance of gestational age at diagnosis and disease type—A single center’s 5 years of experience involving 223 cases

**DOI:** 10.3389/fsurg.2023.1055245

**Published:** 2023-02-15

**Authors:** Xinrui Yang, Weiran Zheng, Xiaoyu Wei, Jingmei Ma, Jie Yan, Liona C. Poon, Huixia Yang

**Affiliations:** ^1^ Department of Obstetrics and Gynecology, Peking University First Hospital, Beijing, China; ^2^ Department of Obstetrics and Gynaecology, Prince of Wales Hospital, The Chinese University of Hong Kong, Hong Kong SAR, China

**Keywords:** cesarean scar pregnancy, gestational week, type, methotrexate, uterine artery embolization, hysteroscopy, prognosis, blood loss

## Abstract

**Objective:**

This study aims to evaluate the importance of the gestational age at diagnosis and the types of cesarean scar pregnancy (CSP) for treatment outcomes and to identify the optimal treatment based on both the gestational age at diagnosis and the CSP type.

**Methods:**

A retrospective cohort study included 223 pregnant women diagnosed with CSP at Peking University First Hospital, Beijing, China, between 2014 and 2018. All CSP cases underwent ultrasound-guided vacuum aspiration followed by supplementary curettage. Adjuvant treatment modalities included intramuscular injection of systemic methotrexate, uterine artery embolization, and hysteroscopy before ultrasound-guided vacuum aspiration. Linear regression was used to determine the relationship between intraoperative blood loss and gestational age at diagnosis, CSP type, highest β-human chorionic gonadotropin level, and management procedures.

**Results:**

None of the patients required blood transfusions or hysterectomies. Patients presenting at <8, 8–10, and >10 weeks had median estimated blood loss of 5, 10, and 35 ml, respectively. Patients with type I CSP, type II CSP, and type III CSP had median blood loss of 5, 5 and 10 ml, respectively. Multivariate linear regression analysis demonstrated that the gestational age at diagnosis (*p* < 0.001) and type of CSP (*p* = 0.023) were independent predictors of intraoperative estimated blood loss. For type I CSP patients, ultrasound-guided vacuum aspiration followed by supplementary curettage alone was performed in 15 of 34 (44.1%) patients, including 12/27 (44.4%) diagnosed at <8 weeks, 2/6 (33.3%) at 8–10 weeks, and 1/1 for >10 weeks. In type II CSP patients, fewer cases were managed by ultrasound-guided vacuum aspiration followed by supplementary curettage alone as the gestational age at diagnosis increased [18/96 (18.8%) for <8 weeks, 7/41 (17.1%) for 8–10 weeks, none for >10 weeks]. Most type III CSP patients (41/45, 91.1%) needed treatments in addition to the ultrasound-guided vacuum aspiration regardless of the gestational age at diagnosis. All CSP patients were treated successfully and did not require readmission or further medical interventions.

**Conclusion:**

Gestational age at diagnosis of CSP and its type show a strong correlation with estimated blood loss during ultrasound-guided vacuum aspiration. With careful management, CSPs may be treated at any gestational week, regardless of their type, with minimal intraoperative bleeding.

## Introduction

Cesarean scar pregnancy (CSP) is a unique type of ectopic pregnancy in which the gestational sac implants at the site of a previous cesarean section scar (1). The overall frequency of CSP in pregnant women is rare and estimated to be approximately 0.05% (2). This rate increases to approximately 2% in women with a history of cesarean section, constituting 4% of all ectopic pregnancies (3). However, the rate of CSP has been gradually rising due to the increasing number of cesarean sections in recent years, together with the implementation of the two-baby policy in China since 2015 (4–7). Patients with CSP are at an increased risk of complications, including excessive bleeding during ultrasound-guided vacuum aspiration (8). If pregnancy is not terminated, patients with CSP are more likely to develop placenta accreta spectrum disorders, one of the most serious adverse outcomes in obstetrics (9, 10).

Dilation and curettage (D&C) with or without ultrasound guidance is associated with a high risk of complications in CSP (11). Grechukhina et al. reported 26 patients with CSP and found that only timely diagnosis and skilled multidisciplinary teamwork could lead to safer outcomes (12).

**Figure 1:**
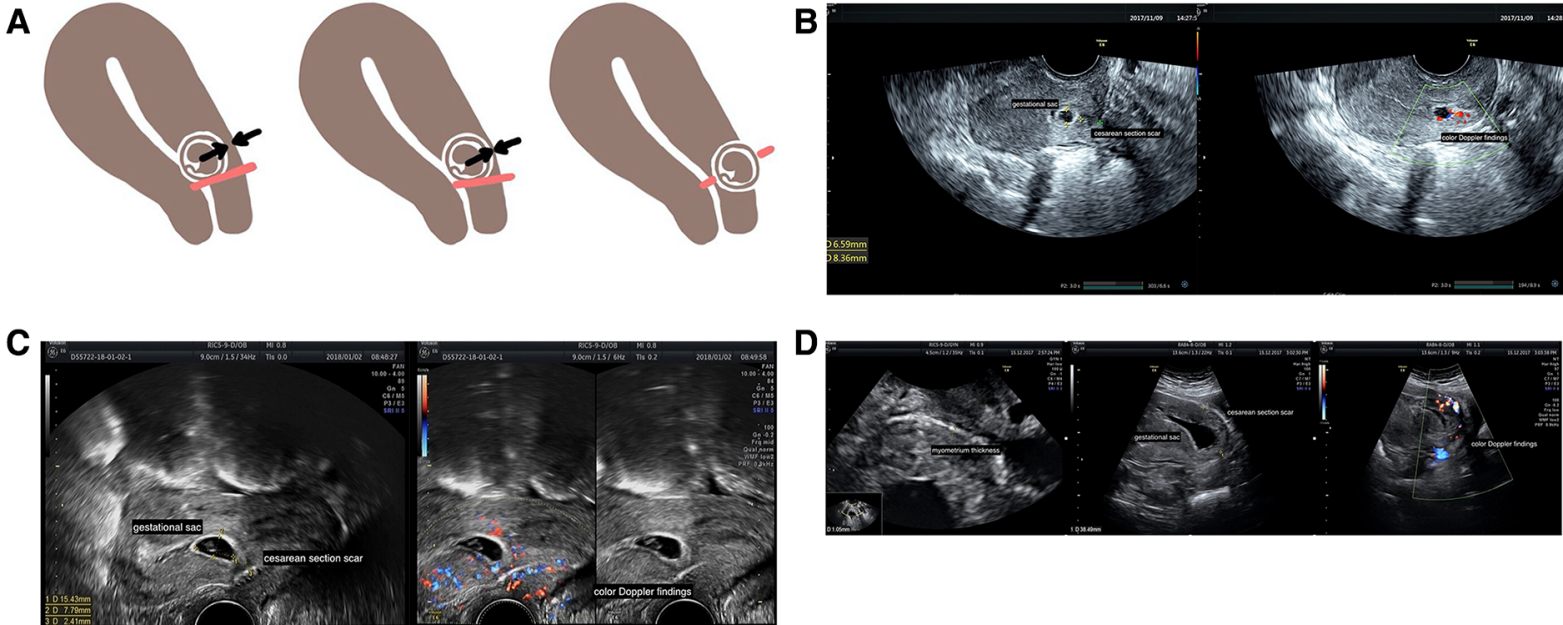
Types of CSP and their ultrasound images. (**A**) Different types of CSP. Red lines refer to the cesarean scar, black or white arrows show the thickness of the myometrium, and brown circles show gestational sacs. Type I CSP is defined as a gestational sac partially located in the scar area having a myometrium thickness of >3 mm. Type II CSP is defined as a gestational sac partially located in the scar area having a myometrium thickness of ≤3 mm. Type III CSP is defined as a gestational sac completely located within the area of the scar having a myometrium thickness of ≤3 mm. Blood flow, illustrated by color flow Doppler, must be observed in the scar area. (**B**) Type I CSP at 7 weeks under transvaginal ultrasound. A gestational sac of approximately 8 mm × 7 mm × 7 mm was partially located in the previous cesarean scar with a myometrium thickness of 8.4 mm. (**C**) Type II CSP at 7 weeks under transvaginal ultrasound. A gestational sac of approximately 15 mm × 11 mm × 8 mm was partially located in the previous cesarean scar with a myometrium thickness of 2.4 mm. (**D**) Type III CSP at 8 weeks under transvaginal ultrasound. A gestational sac of approximately 30 mm × 40 mm × 24 mm that is completely located within the previous cesarean scar with a myometrium thickness of 1.05 mm. CSP, cesarean scar pregnancy;

According to diagnostic criteria used in China based on ultrasonography, CSP is divided into three types, mainly according to the location of the gestational sac and the minimum thickness of the myometrium (13). Type I CSP is defined as a gestational sac partially located in the scar area having a myometrium thickness of >3 mm. Type II CSP is defined as a gestational sac partially located in the scar area of the scar having a myometrium thickness of ≤3 mm. Type III CSP is defined as a gestational sac completely located in the scar area having a myometrium thickness of ≤3 mm (Figure 1). Blood flow, as detected by color flow Doppler, must be observed in the scar area (13). With Doppler ultrasound, CSP can be detected by transvaginal ultrasonography and treated early. Rotas et al. (14) reported that the accuracy of diagnosis was as high as 86.4%. Another prospective study including 485 cases of suspected CSP demonstrated that transvaginal ultrasonography achieved a high level of accuracy with detection rates of 82.4%, 80.6%, and 95.2% for type I, II, and III CSPs, respectively (4).

Although there are several treatment options for managing CSP, their indications and outcomes according to gestational age at diagnosis and CSP type are unclear. In our study, we aimed to evaluate the importance of gestational age at diagnosis and CSP type on treatment outcomes and establish guidelines for selecting a treatment strategy. We also aimed to identify optimal treatment based on both the gestational age at diagnosis and CSP type.

## Materials and methods

This was a retrospective cohort study of 223 confirmed cases of CSP out of 253 suspected cases of CSP treated at Peking University First Hospital, Beijing, China, between January 1, 2014, and December 31, 2018. Thirty patients were excluded from the present study for the following reasons: seven patients with type I to II CSP continued pregnancy under careful monitoring and resulted in a live birth. Three patients terminated pregnancy by a cesarean section or induced labor at 12–20 weeks for vaginal bleeding and abdominal pain, with intraoperative bleeding of 300–500 ml. Others underwent laparoscopic operation due to clinical manifestations, including vaginal bleeding, abdominal pain and anemia, lack of myometrium larger than 5 mm × 5 mm, and abundant blood flow under color Doppler, which were considered difficult for hemostasis under hysteroscopy, with intraoperative bleeding of 5–50 ml. This study was approved by the ethics committee of Peking University First Hospital (ID:2016[1266]), and it was a secondary analysis of existing data. Because this is a research hospital, patients are notified that their clinical data might be used for future data analysis in the admission notification. All patients signed informed consent forms for future data analysis at the time of admission to the hospital. Inclusion criteria are as follows: (1) a transvaginal ultrasound was undertaken to confirm the diagnosis of CSP at our hospital before an ultrasound-guided vacuum aspiration procedure; (2) a history of previous cesarean section; and (3) documentation of gestational age at diagnosis of CSP. Exclusion criteria are as follows: (1) spontaneous miscarriage without the need for surgical intervention; (2) missing operative data; (3) reexamination that showed no evidence of CSP; and (4) surgical repair of the cesarean section scar using laparoscopic/transabdominal approaches immediately after the ultrasound-guided vacuum aspiration procedure as part of a single surgery (following consultation with the attending physicians). Data on the highest β-human chorionic gonadotropin (β-hCG) level, administration of systemic methotrexate (MTX), performance of uterine artery embolization (UAE), and hysteroscopy before the ultrasound-guided vacuum aspiration procedure were collected.

### Diagnosis of cesarean scar pregnancy

The diagnosis of CSP was based on the following transvaginal ultrasound scan findings: (1) an empty uterine cavity; (2) a clearly visible empty endocervical canal without direct contact with the gestational sac; (3) the presence of a gestation sac, with or without a fetal pole, with or without fetal cardiac activity (depending on the gestational age), in the anterior part of the uterine isthmus on the prior cesarean section scar; and (4) the absence of or a defect in the myometrial tissue between the bladder and the gestational sac (15, 16). All transvaginal ultrasound reports were written and reviewed by the same group of doctors experienced in diagnostic medical sonography, and the diagnosis of CSP was stratified into three types, as mentioned above (13).

### Management of cesarean scar pregnancy

All CSP cases were treated by ultrasound-guided vacuum aspiration followed by supplementary curettage. Adjuvant treatment modalities included systemic administration of MTX at a dose of 50 mg/m^2^ by intramuscular injection (17), UAE, and hysteroscopy before ultrasound-guided vacuum aspiration. Choices of adjuvant treatment were made according to the advice of the attending physician in discussion with the patients. Informed consent for treatments was acquired from each patient. Patients with type II or type III CSP frequently received systemic MTX before the operation; UAE was recommended for cases with abundant blood flow surrounding the gestation sac, identified by Doppler ultrasound, or when the gestational age was more than 8 weeks. When the remaining uterine myometrium was considered weak or a niche was suspected to be formed, hysteroscopy was used for detailed examination. MTX and UAE were administered within 24–72 h before the operation.

Experienced gynecological surgeons performed ultrasound-guided vacuum aspiration, while the same group of doctors experienced in diagnostic medical sonography performed the scan. Ultrasound was performed throughout the entire operation. First, the gestation sac was located by ultrasound, followed by careful vacuum aspiration. The size of the metal suction tube was determined based on the gestational week. The implanted site of the gestational sac was gently curetted to ensure complete removal of the gestational tissue.

Blood loss was estimated by suction and weighing of swabs. The amount of bleeding during the operation was considered an important indicator of the outcome to measure the effectiveness of each method. The estimated blood loss was compared according to the gestational age at diagnosis and the type of CSP. Intraoperative bleeding greater than 20 ml was considered significant; regular ultrasound-guided vacuum aspiration followed by supplementary curettage only caused 2–5 ml of intraoperative bleeding in our hospital. When there was active bleeding immediately after ultrasound-guided vacuum aspiration operation or the intraoperative blood loss was greater than approximately 50 ml, uterine tamponade strategies such as intrauterine balloon were employed. For this purpose, a Foley catheter was inserted into the uterine cavity under ultrasound guidance at the end of the operation and the balloon was inflated to 5–50 ml in volume until active bleeding ceased.

All patients received routine follow-up care at the clinic approximately 1 month after the operation.

### Statistical analysis

Descriptive data are presented as counts (percentages) or medians (interquartile ranges) after testing for normality. The Mann–Whitney *U* test or Kruskal–Wallis test was used to compare differences between medians. Forward multivariate linear regression analysis was performed to determine the relationship between intraoperative bleeding and the gestational age at diagnosis, the CSP type, the highest β-hCG level, and the management procedure. Binary logistic regression was used to evaluate which factors among the gestational age at diagnosis, the CSP type, the highest β-hCG level, and the management procedure were independent predictors for significant intraoperative bleeding. A two-tailed *p* value less than 0.050 was considered statistically significant in both univariate and multivariate analyses.

The Statistical Package for Social Science (SPSS) version 24.0 was used for statistical analysis.

## Results

The median age was 35 years (range 24–46 years), the median gravidity was 3 (range 1–9), and the median parity was 1 (range 1–3). Thirty-nine (17.5%) cases had two or more previous cesarean sections, while 110 (49.3%) cases had two or more surgical terminations of pregnancy. A total of 145 (65.0%), 64 (28.7%), and 14 (6.3%) patients presented at <8, 8–10,, and >10 weeks, respectively. A total of 34 (15.3%), 141 (63.2%), and 45 (20.2%) patients were diagnosed with type I, type II, and type III CSP, respectively, while 3 (1.3%) patients were unclassified due to the lack of myometrial thickness in their reports. These three patients with unclassified CSP diagnoses were excluded from further analysis that required precise information about the gestational age and CSP type. None of the patients received a blood transfusion or hysterectomy. In our study, 39 patients (17.5%) required intrauterine tamponade to achieve adequate hemostasis. All the treatments we used are listed in Table 1. All patients were treated successfully without readmission or further medical intervention and had an average hospital stay of 3.579 ± 1.929 days, with a maximum of 16 days. Thirty-eight (17.1%) patients were confirmed to have fetal arrest by ultrasound before the operation.

**Table 1:** Treatment modalities employed for cesarean scar pregnancy.*

Treatment options	*n* (%)	Gestational age (weeks)^a^	Type (*n*)		
All	223 (100)	7 (6–8)	I (*n* = 34)	II (*n* = 141)	III (*n* = 45)
Ultrasound-guided vacuum aspiration only	44 (19.7)	7 (6–8)	8 (23.5)	23 (16.3)	12 (26.7)
MTX, ultrasound-guided vacuum aspiration	105 (47.1)	7 (6–8)	16 (47.1)	73 (51.8)	14 (31.1)
UAE^b^, ultrasound-guided vacuum aspiration	26 (11.7)	7 (6–8)	3 (8.8)	16 (11.3)	7 (15.6)
Hysteroscopy, ultrasound-guided vacuum aspiration	3 (1.35)	9 (7–)	0	2 (1.4)	1 (2.22)
MTX, UAE, ultrasound-guided vacuum aspiration	24 (10.8)	7 (6–8)	5 (14.7)	15 (10.6)	4 (8.89)
UAE, hysteroscopy, ultrasound-guided vacuum aspiration	16 (7.17)	7 (6–8)	2 (5.9)	9 (6.4)	5 (11.1)
MTX, hysteroscopy, ultrasound-guided vacuum aspiration	2 (0.90)	9 (7–)	0	1 (0.7)	1 (2.22)
MTX, UAE, hysteroscopy, ultrasound-guided vacuum aspiration	3 (1.35)	7 (7–7)	0	2 (1.4)	1 (2.22)

MTX, methotrexate; UAE, uterine artery embolization.

^a^ Median (interquartile range).

^b^ Uterine artery embolism.

*Intrauterine balloon was inserted at the end of the operation when there was excessive blood loss.

### Clinical characteristics and estimated blood loss of CSP patients

Patients presenting at <8, 8–10, and >10 weeks had median estimated blood loss of 5, 10, and 35 ml, respectively. Patients with type I CSP, type II CSP, and type III CSP had median blood loss of 5, 5, and 10 ml, respectively (Table 2). The median estimated blood loss for all 223 patients was 5 (5–20) ml.

**Table 2:** Univariate and multivariate analyses of clinical characteristics and management in predicting estimated blood loss in patients with cesarean scar pregnancy.

		*n*	Estimated blood loss (ml)^a^	Univariate analysis		Multivariate analysis	
				U/K/beta	*p* value	Beta	*p* value^b^
Gestational age in weeks	<8	145	5 (5–10)	15.268	0.000^c^	0.247	0.000
	8–10	64	10 (5–20)				
	>10	14	35 (8.75–100)				
Type of CSP^d^	Type I	34	5 (5–10)	11.540	0.003^c^	0.157	0.023
	Type II	141	5 (5–10)				
	Type III	45	10 (5–50)				
Highest β-hCG^e^		223	5 (5–20)	0.076	0.270^b^	0	0
Administration of MTX before operation	No	89	5 (5–10)	0		0	
	Yes	134	10 (5–20)	4,126.000	0.000^f^	−0.029	0.672
Uterine artery embolism	No	154	5 (5–20)	0		0	
	Yes	69	5 (5–10)	4,752.500	0.181^f^	0	0
Hysteroscopy	No	199	5 (5–20)	0		0	
	Yes	24	5 (5–10)	1,758.500	0.025^f^	−0.677	0.499

CSP, cesarean scar pregnancy; MTX, methotrexate; β-hCG, β-human chorionic gonadotropin.

^a^ Median (interquartile range).

^b^ Linear regression.

^c^ Kruskal–Wallis test.

^d^ Cesarean scar pregnancy.

^e^ Human chorionic gonadotrophin.

^f^ Mann–Whitney U test.

Univariate regression analysis demonstrated that gestational age at diagnosis (*p* < 0.001), type of CSP (*p* = 0.003), use of systemic MTX before operation (*p* < 0.001), and hysteroscopy (*p* = 0.025) were predictors of estimated blood loss during ultrasound-guided vacuum aspiration followed by supplementary curettage but not the highest β-hCG level nor UAE (*p* > 0.050). Multivariate linear regression analysis demonstrated that gestational age at diagnosis (*p* < 0.001) and type of CSP (*p* = 0.023) were independent predictors of intraoperative estimated blood loss (Table 2).

We selected the gestational age at diagnosis and CSP type and performed a binary logistic regression using 20 ml as the cutoff for significant intraoperative bleeding. Gestational age at diagnosis >10 weeks was associated with an increased risk of significant intraoperative bleeding [odds ratio (OR) 6.619, 95% CI 2.069–21.180, *p* = 0.001] compared with gestational age <8 weeks. Similarly, type III CSP was associated with an increased risk of significant intraoperative bleeding (OR 3.733, 95% CI 1.294–10.774, *p* = 0.015) compared with type I CSP (Table 3).

**Table 3:** Binary logistic regression for the prediction of significant intraoperative blood loss according to the gestational age and cesarean scar pregnancy type.

	B	*p*	OR	95% CI		B	*p*	OR	95% CI
<8 weeks		0.005			Type I		0.011		
8–10 weeks	0.43999	0.196	1.553	0.797–3.026	Type II	0.35482	0.471	1.426	0.544–3.739
>10 weeks	1.89000	0.001	6.619	2.069–21.180	Type III	1.31730	0.015	3.733	1.294–10.774

OR, odds ratio.

### Different treatment options according to the gestational age at diagnosis and type of CSP

We used the gestational age at diagnosis and CSP type to evaluate the treatment modalities we applied to the CSP patients (Table 4). For type I CSP patients, 15 out of 34 (44.1%) patients received ultrasound-guided vacuum aspiration followed by supplementary curettage as the only treatment modality, and this included 12/27 (44.4%) diagnosed at <8 weeks, 2/6 (33.3%) at 8–10 weeks, and 1/1 for >10 weeks. The median estimated blood loss was 5 ml (*p* = 0.716) in type I CSP patients diagnosed at <8 weeks, regardless of whether they were treated with ultrasound-guided vacuum aspiration followed by supplementary curettage only (*n* = 12) or with other treatment methods (*n* = 15).

**Table 4:** Different management strategies according to the gestational age at diagnosis and cesarean section pregnancy type.

	Type I			Type II			Type III			Total		
	*n*	Treatments	Bleeding^a^	*n*	Treatments	Bleeding^a^	*n*	Treatments	Bleeding^a^	*n*	Treatments	Bleeding^a^
<8 weeks	12	Vacuum aspiration only	5 (5–8.75)	18	Vacuum aspiration only	5 (5–22.5)	2	Vacuum aspiration only	4, 5	32	Vacuum aspiration only	5 (5–10)
	15	Others^b^	5 (5–10)	52	MTX	10 (5–17.5)	10	MTX	20 (5–27.5)	72	MTX	10 (5–20)
				26	Others	5 (4.75–6.25)	7	Others	5 (5–10)	38	Others	5 (5–6.25)
8–10 weeks	2	Vacuum aspiration only	4, 20	7	Vacuum aspiration only	5 (5–10)	2	Vacuum aspiration only	10, 200	11	Vacuum aspiration only	5 (5–10)
	3	MTX	5, 5, 20	19	MTX	10 (5–30)	6	MTX	12.5 (5–31.25)	28	MTX	10 (5–10)
	1	UAE	10	15	Others	10 (5–20)	9	Others	10 (5–85)	25	Others	10 (5–10)
>10 weeks	1	Vacuum aspiration only	5	1	MTX	50	2	MTX	100, 500	1	Vacuum aspiration only	5
				1	UAE	3	2	UAE	5, 10	3	MTX	50,100,500
				2	Others	10,50	5	Others	20, 20, 50, 100, 300	10	Others	20 (8.75–62.5)
Total	15	Vacuum aspiration only	5 (5–10)	25	Vacuum aspiration only	5 (5–10)	4	Vacuum aspiration only	4, 5, 10, 200			
	19	Others	5 (5–10)	72	MTX	10 (5–20)	18	MTX	20 (5–50)			
				44	Others	5 (5–10)	23	Others	10 (5–50)			

MTX, methotrexate; UAE, uterine artery embolization.

Green: safe to perform ultrasound-guided vacuum aspiration alone. Yellow: better use UAE or hysteroscopy with or without MTX, especially for patients with color Doppler findings in our clinical works. Orange: none of the methods were superior to others, and MTX was better combined with UAE or hysteroscopy, as determined by the patient’s condition, especially for patients >10 gestational weeks. Dots: lack of cases, without enough statistical evidence and considered empirically.

^a^ Median (interquartile range), if the sample capacity is less than 5, only numbers.

^b^ Other treatments include at least one uterine artery embolism or hysteroscopy.

In type II CSP patients, the percentage of patients managed by ultrasound-guided vacuum aspiration followed by supplementary curettage as the sole treatment modality reduced as the gestational age at diagnosis increased [18/96 (18.8%) for <8 weeks, 7/41 (17.1%) for 8–10 weeks, none for >10 weeks]; therefore, we compared the estimated blood loss between MTX and other treatment methods. In type II CSP patients diagnosed at <8 weeks, additional treatments including UAE (*n* = 24) or hysteroscopy (*n* = 2) prior to ultrasound-guided vacuum aspiration were associated with a lower estimated blood loss in comparison to systemic MTX prior to ultrasound-guided vacuum aspiration (5 vs. 10 ml for median bleeding, *p* = 0.002) but not for type II CSP patients diagnosed at 8–10 weeks (median bleeding 10 vs. 10 ml, *p* = 0.857). There were only four cases of type II CSP patients diagnosed >10 weeks. One patient who underwent UAE before ultrasound-guided vacuum aspiration had an estimated blood loss of 3 ml, one patient who underwent hysteroscopy plus UAE before ultrasound-guided vacuum aspiration had an estimated blood loss of 10 ml, and one patient who underwent systemic MTX plus UAE before ultrasound-guided vacuum aspiration had an estimated blood loss of 50 ml.

Most type III CSP patients (41/45, 91.1%) needed additional interventions in addition to ultrasound-guided vacuum aspiration regardless of the gestational age at diagnosis, and MTX (*n* = 18) or other treatment methods (*n* = 23) demonstrated no significant differences in estimated intraoperative blood loss (20 vs. 10 ml for median bleeding, *p* = 0.528).

## Discussion

Our study has shown that the gestational age at diagnosis and type of CSP are predictors of intraoperative estimated blood loss in patients with CSP but not the highest β-hCG level, systemic use of MTX, UAE, or hysteroscopy.

Management of CSP is largely based on expert opinions rather than standardized guidelines (13). The treatment decision for each CSP patient was mainly based on gestational weeks and CSP types, as well as color flow Doppler demonstrating blood flow around the gestational sac.

Several methods could potentially reduce bleeding or reduce embryonic activity before or during the operation. Successful treatment of CSP using systemic MTX was reported (17). As there were no clear data about the effect of UAE on future reproductive outcomes, the Society of Interventional Radiology (SIR) did not consider UAE to be a relative contraindication when the patient desires to maintain childbearing potential and thus encouraged its usage (18). MTX, UAE, and hysteroscopy have frequently been used, either alone or in combination, under different clinical situations prior to ultrasound-guided vacuum aspiration (15, 19, 20). Sorrentino et al. also raised a new idea of treating CSP by UAE combined with a hysteroscopic diode laser (21).

Gestational age at diagnosis was the first consideration when deciding treatment options, followed by the type of CSP. In our study, we divided the CSP cases into three gestational age groups: <8 gestational weeks, 8–10 gestational weeks, and >10 gestational weeks. Incidentally, the blood β-hCG level reaches its highest level at 8–10 gestational weeks. Our findings that the estimated blood loss increases with increasing gestational age at diagnosis of CSP are not surprising. According to a study by Wu et al. (22), gestational age at diagnosis of <7 weeks was a favorable factor for successful treatment with ultrasound-guided vacuum aspiration followed by supplementary curettage alone, defined as normal serum β-hCG levels without a CSP mass, residual pregnancy tissue, or major complications requiring further treatment. We did not expect excessive blood loss during ultrasound-guided vacuum aspiration followed by supplementary curettage when the CSP diagnosis was made before 7 weeks because pregnancy contents are loosely attached to the uterine wall at this stage, according to Hoshino et al. (23).

The CSP classification that we used in this study is widely applied in our clinical practice. It has been advised that all type II and type III CSP patients and type I CSP patients with diagnoses made after 8 gestational weeks should receive MTX treatment or undergo UAE before ultrasound-guided vacuum aspiration (13). In our study, we found that in patients with type I CSP diagnosed at <8 weeks, there was no significant difference in estimated blood loss between those treated with ultrasound-guided vacuum aspiration followed by supplementary curettage alone and those treated in combination with other modalities including UAE and hysteroscopy. In contrast, these additional modalities should be considered for type II and type III CSP. Most type III CSP patients could not be managed with ultrasound-guided vacuum aspiration alone. Neither MTX nor other treatment methods show advantages in reducing intraoperative bleeding in type III CSP patients; therefore, a combination of MTX with UAE or hysteroscopy might be needed for this group of patients. Wei and Yu demonstrated that, in type II and III CSP patients within 7 gestational weeks, UAE was not associated with reduced bleeding when compared to treatment with MTX and suggested that MTX could be used in type II and III CSP patients within 7 gestational weeks to avoid the adverse effect of UAE and reduce the length and expenses of hospital stay (16). Zhuang and Huang conducted a prospective trial in 72 cases of CSP diagnosed between 6 and 13 gestational weeks, compared the effects of preoperative MTX and UAE, and found that the MTX group had more bleeding than the UAE group (415.63 vs. 36.93 ml, *p* < 0.001) (19). In our study, UAE was mostly used in combination with MTX or hysteroscopy when there was abundant blood flow surrounding the gestational sac as identified by color Doppler ultrasound. The use of UAE alone before ultrasound-guided vacuum aspiration seemed quite limited, given the gestational age and CSP type. The study did not have sufficient data to compare UAE and systemic MTX.

Unexpectedly, neither the highest β-hCG level, MTX, UAE, nor hysteroscopy was associated with the estimated blood loss in multivariate analysis. Wu et al. also found that preoperative serum β-hCG levels had a limited influence on prognosis (22). Stabile et al. raised the idea that a possible reason for the limited efficacy of systemic MTX as monotherapy is that scar tissue interferes with achieving a sufficient level of MTX near the gestational sac but that this could be overcome by combining with D&C or hysteroscopy (20). Systemic MTX plus ultrasound-guided vacuum aspiration followed by supplementary curettage was used in nearly half of our patients, while UAE and hysteroscopy were performed in combination with MTX before ultrasound-guided vacuum aspiration in most situations. Gestational weeks over 8 weeks and abundant blood flow in Doppler ultrasound would also indicate active preparative management such as systemic MTX or UAE.

Based on the results of estimated blood loss according to the different treatment options (Table 4), we propose that for type I CSP, the patients can be managed by ultrasound-guided vacuum aspiration followed by supplementary curettage as a single treatment modality because even patients with over 8 gestation weeks did not bleed more than 20 ml if they were diagnosed with type I CSP (seven cases). For type II and type III CSP diagnosed at <8 weeks, we propose treatments combined with UAE or hysteroscopy rather than MTX injection; only 20/115 (17.4%) of these patients could be managed by ultrasound-guided vacuum aspiration followed by supplementary curettage alone. Finally, for type II and type III CSP diagnosed at ≥8 weeks, we propose to employ MTX injection in combination with UAE or hysteroscopy based on each patient’s condition; these patients could be seldom managed by ultrasound-guided vacuum aspiration followed by supplementary curettage alone (9/71, 12.7%), and none of the treatments seemed safe enough as a single therapy. Ultrasonic diagnosis was not the sole factor in selecting treatment strategies. In our clinical practice, MTX or other treatments were used more frequently as the gestational week progressed. With careful management and patient selection, we were able to treat patients with a relatively low level of intraoperative bleeding (a median of 5 ml) at all gestational weeks or with all CSP types. It seemed that our intraoperative blood loss was less than that in other centers (1, 22); this may be because we were carefully selecting patients with complex situations for laparoscopic operations.

Our study was the first to evaluate the importance of gestational age at diagnosis and CSP type for predicting intraoperative bleeding. The limitations of this study included the following: first, this study was a retrospective study, and the results could be biased compared to a prospective study. Second, the small sample size in each CSP type group and gestational age group may have reduced the power of our study. Third, we used 20 ml as a cutoff for significant bleeding in our center, but this may not be applicable to other hospitals. The management of CSP needs further investigation, preferably with a prospective randomized trial comparing the modalities of MTX, UAE, and hysteroscopy in CSP patients according to the gestational age at diagnosis and CSP type. Studies that focus on cesarean scar repair procedures are also needed.

## Conclusion

Our study has demonstrated that gestational age at diagnosis of CSP and its type both show a strong influence on estimated blood loss during the ultrasound-guided vacuum aspiration operation. With careful management, any gestational week or type could be treated with a relatively low intraoperative bleeding. It appears safe for CSP patients of any gestational week with type I CSP to undergo ultrasound-guided vacuum aspiration followed by supplementary curettage as a single therapy. Preoperative adjuvant treatments, either UAE or hysteroscopy, might be needed for type II and type III CSP patients diagnosed at <8 weeks by color Doppler ultrasonography. For type II and type III CSP patients over 8 weeks, a combination of systemic MTX with UAE or hysteroscopy might need to be considered.

## Data Availability

The raw data supporting the conclusions of this article will be made available by the authors, without undue reservation.
